# Genomic Evolution and Surveillance of Respiratory Syncytial Virus during the 2023–2024 Season

**DOI:** 10.3390/v16071122

**Published:** 2024-07-12

**Authors:** Madeline Yunker, Amary Fall, Julie M. Norton, Omar Abdullah, David A. Villafuerte, Andrew Pekosz, Eili Klein, Heba H. Mostafa

**Affiliations:** 1 Johns Hopkins School of Medicine, Department of Pathology, Division of Medical Microbiology, Meyer B-121F, 600 N. Wolfe St., Baltimore, MD 21287, USA; myunker1@jhmi.edu (M.Y.); afall2@jhmi.edu (A.F.); jnorto19@jhmi.edu (J.M.N.); oabdull1@jhmi.edu (O.A.); dvillaf2@jh.edu (D.A.V.); 2 Department of Emergency Medicine, Johns Hopkins School of Medicine, Baltimore, MD 21287, USA; apekosz@jhsph.edu (A.P.); eklein@jhu.edu (E.K.); 3 W. Harry Feinstone Department of Molecular Microbiology and Immunology, The Johns Hopkins Bloomberg School of Public Health, Baltimore, MD 21287, USA; 4 Center for Disease Dynamics, Economics, and Policy, Washington, DC 20005, USA

**Keywords:** respiratory syncytial virus, RSV, surveillance, genomic sequencing

## Abstract

Respiratory syncytial virus (RSV) is a significant cause of morbidity, particularly in infants. This study describes RSV genomic diversity and disease outcomes during the 2023–2024 season in the Johns Hopkins Hospital System (JHHS). Between August and December 2023, 406 patient samples were sequenced, showing that RSV-B GB5.0.5a was the dominant genotype detected. RSV-A genotype GA2.3.5 was detected less frequently. Metadata analysis of patient data revealed that, although RSV-B was more commonly detected, patients with RSV-A infections were more frequently hospitalized. Analysis of both the G- and F-genes revealed multiple amino acid substitutions in both RSV-A and RSV-B, with some positions within the F-protein that could be associated with evasion of antibody responses. Phylogenetic analysis revealed the genetic diversity of circulating GB5.0.5a and GA2.3.5 genotypes. This study serves as an important baseline for genomic surveillance of RSV within the JHHS and will assist in characterizing the impact of the newly approved RSV vaccines on RSV genomic evolution and the emergence of escape mutations.

## 1. Introduction

Respiratory syncytial virus (RSV) is a significant cause of lower respiratory tract infections, causing bronchiolitis and pneumonia in infants and young children globally [1,2]. By the age of one, approximately 60–70% of children are infected with RSV, with 2–3% requiring hospitalization [3]. RSV seasonality is largely dependent on geographical location and generally results in annual epidemics during the winter months in temperate climates and year-round transmission in (sub)tropical climates [1,4]. However, the timing and duration of epidemics can vary widely between and within countries [1,5]. RSV activity also changes at the local level, with season onset, peak, and decline varying between 0 and 5 weeks across the country, within the same state [5,6], and in tropical countries [5]. Although circulating genotypes can be similar between two neighboring cities, genetic variation between locations is notable [7]. This indicates that local surveillance systems can be valuable for understanding surges in disease, outbreaks, and seasonality [5]. The infrequency of RSV genomic and clinical data largely stems from immature surveillance systems, which mainly rely on pre-existing influenza surveillance [3,4]. Genomic RSV surveillance will facilitate an understanding of outbreaks, informed responses, and the ability to predict future epidemiological waves [4]. Additionally, baseline RSV genome characterization is expected to facilitate future investigations on the impact of the recently approved RSV vaccines on RSV genomic evolution and changes in associated disease severity. In this study, we characterized RSV genotypes of patients diagnosed at the Johns Hopkins Health System (JHHS) during the 2023/2024 season and provided a description of the associated clinical presentations and outcomes.

## 2. Materials and Methods

### 2.1. Study Population

Standard-of-care diagnostic RSV testing was conducted for both inpatients and outpatients across JHHS hospitals and outpatient practices. Testing for RSV A/B was performed with Cepheid (Sunnyvale, CA, USA) Xpert Xpress SARS-CoV-2/Flu/RSV test or Roche (Basel, Switzerland) ePlex Respiratory Pathogen Panels [8,9]. Overall, 52,343 tests were conducted for RSV between June 2023 and February 2024. Of these, 4.6% (2420/52,343) were positive for RSV. Out of the 2420 samples positive for RSV, 17.2% (417/2420) were randomly selected (convenience sample, based on availability, sufficient volume, and proper storage after the standard-of-care testing) for genome sequencing. Study samples were collected between August 2023 and December 2023. Clinical and demographic data were collected in bulk from the electronic health record systems as described previously [10]. RSV-related admissions were defined based on laboratory testing results: patients tested with the extended respiratory panel were assumed to be symptomatic, and patients who tested positive only for RSV prior to admission were assumed to be admitted for RSV-related illness. Patients who tested positive for other targets were not included as being admitted for RSV-related illness.

### 2.2. Nucleic Acid Extraction and Real-Time PCR

Viral nucleic acid was extracted from remnant patient samples using the Chemagic Viral RNA/DNA Kit following the manufacturer’s instructions (Revvity (Waltham, MA, USA)), with 300 μL extracted for each sample and eluted into 60 μL volume. To distinguish between RSV-A and RSV-B and obtain cycle threshold (Ct) values, real-time PCR was performed using the Luna Universal Probe One-Step RT-qPCR Kit per the manufacturer’s guidelines (New England BioLabs, Ipswich, MA, USA). Samples with Ct values 33 and above were excluded from additional analysis.

### 2.3. Whole-Genome Amplification and Sequencing

Whole-genome amplification was adapted from Wang et al. [11] by using 2 μL of first-round PCR products for the nested PCR. Library preparation for sequencing followed the manufacturer instructions of the DNA Native Barcoding Kit 96 v14 (SQK-NBD114.96) for the PromethION and the NEBNext ARTIC Library Prep kit (New England BioLabs/Oxford Nanopore Technologies (Oxford, UK)). The input amount of the PCR product used was 7 μL of the nested PCR product in addition to 4 μL of the first-round PCR product into the end-prep reaction. EDTA was added to the barcoded amplicons to stop the reaction. Samples were pooled and 5 μL/sample AMPure Beads were used for sample cleaning. Elution was added to 10 μL of NEBNext Quick Ligation Reaction Buffer, 5 μL of native adaptor, and 5 μL of Quick T4 DNA Ligase and incubated at room temperature for 20 min. Beads (30 μL) were added, and clean-up steps were repeated using 125 μL of SFB buffer. The library was eluted in 35 μL of elution buffer. The entire 35 μL library was used for sequencing.

### 2.4. Virus Genome Assembly and Phylogenetic Analysis

The resulting fastq files were analyzed using our in-house pipeline. The closest references were selected by blasting against the RSV reference genomes (NC_038235 and NC_001781). Draft genomes were generated by mini_assemble within pomoxis, using medaka consensus to further enhance the draft genome and establish a consensus sequence. Sequencing depth was evaluated with samtools. The alignment of sequences was performed using the built-in pipeline in NextClade. Clades and amino acid substitutions (AASs) were determined by the built-in pipeline in NextClade. Quality control parameters were used to remove sequences of mediocre and bad quality, with scores between 30 and 90+. Genomes were visualized using BioEdit and IGV to assess the presence of gaps and coverage. Sequences with gaps were removed from the analysis. The phylogenetic trees for RSV-A and RSV-B were generated using the maximum likelihood method using IQ-Tree version 2.2.6. The visualization was performed using FigTree version 1.4.4. Complete reference genomes used for the phylogenetic analysis can be found in Supplementary Table S1. The ModelFinder in IQ-TREE2 was used to select the best-fitted nucleotide substitution model. The robustness of the tree topology was tested with 1000 nonparametric bootstrap analyses. Bootstrap values > 75% were shown on branches of the consensus trees.

## 3. Results

### 3.1. RSV Prevalence at JHHS and the Study Cohort

**Figure 1:**
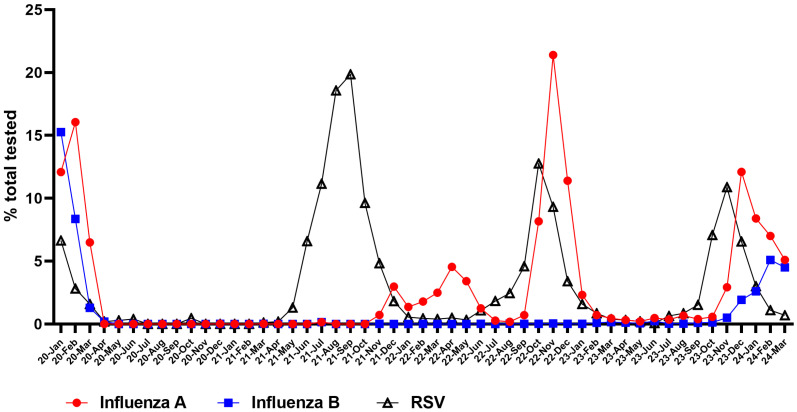
Percent positive tests (%) for influenza A, influenza B, and RSV between January 2020 and December 2023.

The 2023/2024 respiratory viral season (≈12.5% at peak) exhibited a lower RSV positivity rate in the JHHS than the 2022/2023 (≈15% at peak) and 2021/2022 (≈20% at peak) seasons (Figure 1). The 2021/2022 RSV season at JHHS exhibited increasing testing positivity rates in early April 2021, peaking in September 2021 with a testing positivity rate close to 20% (Figure 1). Similarly, the 2022/2023 season exhibited increasing testing positivity rates in June 2022, peaking in October 2022 with a testing positivity rate of 12.8% (Figure 1). For the 2023/2024 season, increased RSV detection started around early September 2023 and peaked in November 2023 with a positivity rate of 10.9% (Figure 1). RSV activity peaked earlier in the season compared to influenza A and B viruses, consistent with the prior two seasons (Figure 1).

Metadata of the 2023/2024 cohort were collected for 406 unique patients. The cohort consisted of slightly more females (51.0%, [207/406]) than males (48.8%, [198/406]) (Table 1). The median age of the cohort was two years old. Children aged 1–5 years were the most represented (45.7%, [183/406]). Infants, 11 months and younger, comprised 29.6% (120/406) of the cohort. Adults 60 years and older (9.1%, [37/406]) and the other age groups were represented to a lesser extent (Table 1). Clinical signs and symptoms were noted from the charts of 89.4% (363/406) of patients at the time of presentation. Cough (32.8%, [119/363]), fever (28.9%, [105/363]), and breathing problems (27.0%, [98/363]), including wheezing and shortness of breath, were most commonly reported (Table 1). One or more comorbidities were noted for 40.1% (163/406) of patients, with cancer (23.9%, [97/406]), immunosuppression (20.7%, [84/406]), and hypertension (17.0%, [69/406]) being most common (Table 1). Admissions were reported for 31.3% (127/406) of the cohort; of those, 23.6% (30/127, and 7.4% of cohort, [30/406]) required ICU-level care (Table 1). Supplemental oxygen was provided to 77.2% (98/127, and 24.1% of cohort [98/406]) of admitted patients (Table 1). The age groups 18–59 years and 60+ years had the highest rates of comorbidities and admissions, as detailed in Table S2. Metadata also revealed 13 (3.2%) patients to have co-infections with rhino/enteroviruses. These co-infections occurred in all age groups, with children 1–5 years of age and infants comprising 46.2% (6/13) and 23.1% (3/13), respectively. Other respiratory pathogens (influenza A/B, parainfluenza 1–4, seasonal coronaviruses, SARS-CoV-2, adenovirus, metapneumovirus, *Chlamydia pneumoniae,* and *Mycoplasma pneumoniae*) were included in the metadata pull but were not detected in our cohort.

Metadata were compared for 374 patients, whose samples were able to be typed for RSV-A, RSV-B, or RSV-A/B (Table 1). RSV-B (51.8%, [146/282]) and RSV-A/B (64.3%, [9/14]) comprised slightly more males than females, whereas the RSV-A cohort had slightly more females (65.4%, [51/78]). The median age for RSV-B, RSV-A, and RSV-A/B were similar. Of note, RSV-A had a higher percentage of adults 18-59 years of age (15.4%, [12/78]) compared to RSV-B (7.4%, [21/282]) and RSV-A/B (7.1%, [1/14]). Clinical signs were reported at the time of presentation in 90.4% (255/282) of RSV-B, 83.3% (65/78) of RSV-A, and 85.7% of RSV-A/B (12/14) and were similar between the three groups. RSV-A had higher admissions than RSV-B, with 42.3% (33/78) of RSV-A and 27.3% (77/282) of RSV-B patients being admitted (Table 1). A total of 27.3% (21/77) of admitted RSV-B patients and 21.2% (7/33) of admitted RSV-A patients required ICU-level care. Rhino/enterovirus co-infections were found in 3.2% (9/282) of RSV-B-positive samples and 3.8% (3/78) of RSV-A-positive samples. No co-infections in RSV-A/B samples were reported.

### 3.2. Genotype Analysis

Following real-time PCR for initial genotyping into RSV-A and RSV-B, 33 (7.9%) samples were excluded from whole-genome sequencing for having Ct values above 33. Of the remaining samples, 289 (69.3%) were identified as RSV-B, 81 (19.4%) were RSV-A, and 14 (3.4%) were RSV-A/B co-detections. A total of 255 (61.2%) sequences had complete G-genes and were assigned GA genotype classifications based on the Nextclade Pipeline. The complete F-gene was recovered from 266 (63.8%) sequences.

### 3.3. G-Gene Analysis

Of the 52 RSV-A samples with G-gene sequences, all (52/52) belonged to G-clade GA2.3.5. Several different clades were identified based on defining AASs in the G-gene (Table 2). A.D.1 comprised 38.5% (20/52), A.D.5.2 was identified in 28.8% (15/52), and A.D.3 in 19.2% (10/52) of samples (Table 2). A.D.2.1 (1.9%, [1/52]), A.D.3.1 (3.8%, [2/52]), and A.D.5.1 (7.7%, [4/52]) were represented to a lesser extent (Table 2).

Of the RSV-B samples with G-gene sequences, all (203/203) belonged to the G-clade GB5.0.5a. Within this clade, three different clades were identified based on defining AASs in the G-gene (Table 2). B.D.E.1 represented 92.1% (187/203) of sequences and B.D.4.1.1 was found in 7.4% (15/203) of samples.

Twelve AASs were identified in the RSV-A G-gene that were found across all or most of the clades when compared to the reference sequence hRSV/A/England/397/2017 (Table 3). P71L, S243I, and I265L were found in 100% of RSV-A genomes. G224E (69.2%), D284G (96.2%), and Y304H (82.7%) were found across all clades but not all samples. H90Y (96.2%, not in A.D.2.1), L101F (88.5%, not in A.D.3.1), I134K (98.1%, not in A.D.2.1), and K262E (98.1%, not in A.D.2.1) were found in four of five clades. Two AASs were found in the majority of samples, T319I (69.2%) and T320A (73.1%) (Table 3). V131D (100%, [10/10]), I141T (70%, [7/10]), H266L (100%, [10/10]), and T136I (90%, [9/10]) were frequently identified within the A.D.3 clade.

Seven AASs were identified to be in most or all the RSV-B clades. A74V (100%), T131A (96.1%), I137T (97.0%), and I252T (90.6%) were found in all RSV-B clades when compared with the reference genome hRSV/B/Australia/VIC-RCH056/2019 (Table 3). Of note, I252T was only found in one (6.7%, [1/15]) B.D.4.1.1 sample. An additional AAS, P289L, was identified across all three clades, but at low percentages (2.5%, [5/203]). P289S (13.3%, [2/15]) was found in clade B.D.4.1.1. The B.D.4.1.1 and B.D.E.1 clades had two additional AASs that were found among the majority of samples. S100G was found in 98.5% (200/203) of the RSV-B samples, and all B.D.E.1 samples contained this substitution (Table 3). P221L was found in 96.6% (196/203) of the RSV-B samples. Three more substitutions were identified in low frequencies between both clades: P101S (B.D.4.1.1: 6.7%, [1/15], B.D.E.1: 1.6%, [3/187]), T141I (B.D.4.1.1: 6.7%, [1/15], B.D.E.1: 0.5%, [1/187]), and E239K (B.D.4.1.1: 13.3%, [2/15], B.D.E.1: 13.9%, [26/187]). A further five AASs were frequently found in B.D.4.1.1 samples: K85E (86.7%, [13/15]), T141A (80%, [12/15]), E224G (80%, [12/15]), P229S (66.7%, [10/15]), and S243I (80%, [12/15]). The loci 85 (K85T) and 141 (T141I) also exhibited substitutions in two samples within the B.D.E.1 clade. Within the B.D.E.1 clade, four AASs were prominent: K256N (90.4%, [169/187]), I268T (98.9%, [185/187]), S275P (98.4%, [184/187]), and Y285H (78.1%, [146/187]). An additional 41 samples (21.9%) had a Y285L substitution.

### 3.4. F-Gene Analysis

The F-genes of 53 (65.4%) RSV-A sequences were examined for AASs. A T122A substitution was found in 43.4% of samples. This locus was not found to have any other substitutions with different amino acid identities within RSV-A samples. A V127I substitution was found in 24.5% of samples (Table 4). A V127A substitution was also found in 9.4% (5/53) of RSV-A samples. The F-genes of 213 (73.7%) RSV-B sequences were examined for AASs. An S211N substitution was detected in 96.2% (205/213) and an S389P substitution was detected in 93.0% (198/213) of RSV-B samples (Table 4). A further 4.2% (9/213) of samples had an S389L substitution.

AASs that have been reported to impact or are at positions with substitutions that have been reported to impact monoclonal antibody binding in both RSV-A and RSV-B F-proteins were detected. An S276N substitution was detected in 5.7% [3/53] of RSV-A samples (Table 5). Additionally, N201K (1.9%, [4/213]), R209Q (2.3%, [5/213]), and R209K (1.4%, [3/213]), which might impact palivizumab and nirsevimab activity, were detected [12].

### 3.5. Phylogeny

**Figure 2:**
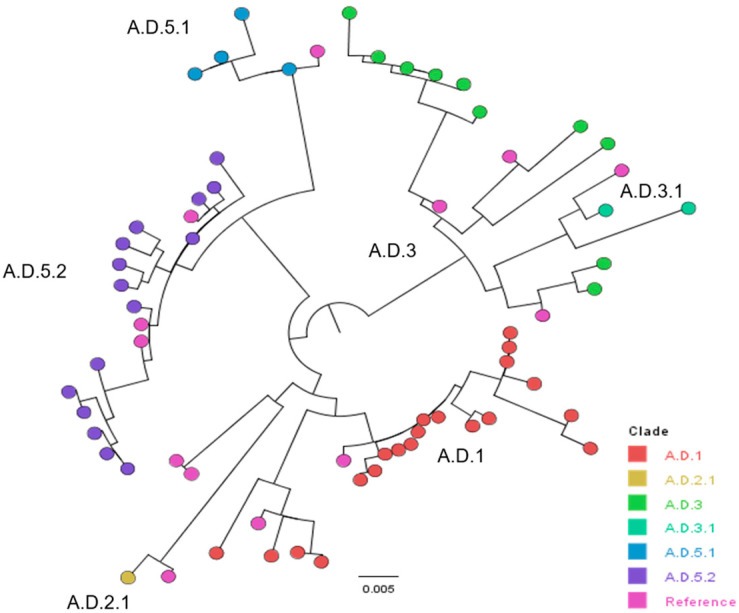
Phylogenetic tree for RSV-A G-gene. Reference genomes are listed in Table S1. All sequences belonged to the G-clade GA2.3.5.

**Figure 3:**
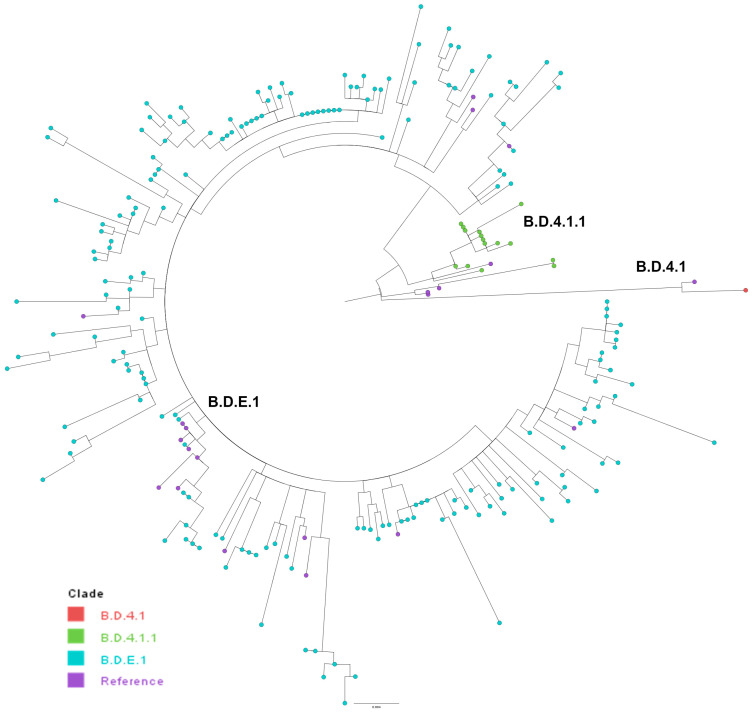
Phylogenetic tree for RSV-B G-gene. All clades belonged to the G-clade GB5.0.5a. Reference genomes are in purple.

The Nextclade G-gene results were confirmed through a phylogenetic analysis for RSV-A (Figure 2) and RSV-B (Figure 3). For RSV-A, the clades described above (A.D.1, A.D.2.1, A.D.3, A.D.3.1, A.D.5.2, and A.D.5.2) clustered together with reference genomes from the GA2.3.5 clade. Each clade clustered with reference genomes from different geographical locations and isolation years (Table S1). The A.D.1 clade had two distinct branches, with reference genomes from the United States but from different years. The smaller cluster was closely related to a 2022 genome, whereas the larger cluster was from 2023. The two A.D.3 samples were found to cluster with the ten A.D.3.1 samples but were found to be within their own branch (Figure 2). Within the A.D.3 genotypes, there were three distinct branches with references from the United States (characterized in 2023 and 2022) and China (characterized in 2019) (Figure 2 and Table S1). The clade A.D.5.1 clustered with a 2022 reference genome from the United States. The largest clade, A.D.5.2, was close to 2022 reference genomes from the United States and two from Germany characterized in 2021 and 2022 (Figure 2 and Table S1). Genotype A.D.2.1 was most closely related to a 2022 reference genome from the United States (Table S1).

For RSV-B, the clades B.D.4.1, B.D.4.1.1, and B.D.E.1 clustered distinctly, confirming the Nextclade analysis (Figure 3). All samples were associated with sequences belonging to the GB5.0.5a genotype. The clade B.D.4.1 clustered distinctly, along with a 2021 reference genome from France (Figure 3 and Table S1). The B.D.4.1.1 clade also clustered distinctly, with some evident diversity. These samples clustered with two geographically and temporally distinct reference genomes. Two samples were most closely related to a 2018 sample from the United States, while the majority clustered with a 2023 sample from Thailand. For B.D.E.1, there were many distinct clusters indicating lots of diversity within this clade. The B.D.E.1 reference genomes were from three different countries in 2021–2024: United States, Thailand, and Germany (Figure 3 and Table S1).

## 4. Discussion

### 4.1. RSV-B Is the Dominant Genotype Characterized from the JHHS

In the years following the emergence of SARS-CoV-2, the seasonality of RSV was temporarily disrupted, as was the case for numerous respiratory pathogens [13]. RSV cases for the 2021/2022 season began in mid-April 2021 and peaked in September. This early beginning to the RSV season was also reported in Washington State [13]. The 2022/2023 season also exhibited an early start, with cases increases starting in July, peaking in October, and ending around February 2023. Washington State, Arizona, and Massachusetts also observed cases increasing and peaking earlier than normal [13,14,15]. Cases during the 2023/2024 season increased in September and peaked in mid-November. This is still earlier than what has been reported in the past, with the United States exhibiting peak RSV season in January/February [4]. Further retrospective studies of RSV circulation within the United States indicated that the season onset usually occurred in October/November, peaking in December/January, and decreasing around March/April [6,16]. However, it is important to note that RSV seasonality is prone to substantial differences at the national and subnational level [5].

The RSV testing positivity rate increased during the 2021/2022 season and decreased during the following two seasons. Our data contrast with those from Washington State, which reported a greater number of cases during the 2022/2023 season [13]. However, an increase in RSV cases in the 2021/2022 and 2022/2023 seasons was observed globally [17,18,19,20]. This increase in cases is thought to be due to reduced protective immunity following the COVID-19 pandemic, with population exposure to RSV having been impacted by non-pharmaceutical interventions (NPIs) [19,21]. Previous studies have shown that in the absence of circulating RSV, antibody titers waned significantly [20]. Studies have indicated reduced anti-RSV antibody levels in infants, women of childbearing age, and in human milk, indicating immune debt [17]. However, another study from Australia showed no differences in the population-level immunity to RSV between pre-pandemic seasons and the 2022 season [20]. It was also hypothesized that a new viral strain with increased fitness may have emerged following the pandemic [13,15]. However, numerous reports from Australia, Japan, Italy, Austria, Argentina, and Spain reported similar genotypes circulating pre- and post-pandemic, indicating that this surge in cases was likely not due to a new viral genotype [7,17,19,20,21,22]. In fact, there was a decrease in the genetic diversity of circulating RSV-A and RSV-B genotypes in Italy and Australia during and after the pandemic, indicating a potential genetic bottleneck introduced due to a sharp reduction in infections [17,20]. The increased number of cases following the pandemic might be attributable to more complex and unknown factors, such as changes in infrastructure, social attitudes, and health-seeking behaviors [20].

RSV-B genotype GB5.0.5a was predominantly (69.5%) detected in our cohort, followed by RSV-A genotype GA2.3.5 (19.2%), and a few co-detections of RSV-A/B (3.4%). In the United States, RSV-A was predominantly detected during the 2021/2022 and 2022/2023 seasons in Washington, Arizona, and Massachusetts [13,14,15]. In Austria and Bulgaria, RSV-B dominated the 2022/2023 season, while RSV-A drove the surge in 2021/2022 [19]. The RSV-B genotype GB5.0.5a comprised all RSV-B samples in Washington State during the previous seasons, whereas RSV-A genotype GA2.3.6b was dominant, with GA2.3.5 circulating to a lesser extent [13]. Similarly, in Arizona, RSV-B genotype GB5.0.5a comprised all RSV-B samples [14]. However, similar to what our data show, RSV-A GA2.3.5 was the only genotype detected. In Massachusetts, all RSV-B belonged to the GB5.0.5a genotype and all RSV-A belonged to the GA2.3.5 genotype [15]. The GB5.0.5a and GA2.3.5 genotypes have been circulating globally since 2017 and reported to be dominant following the SARS-CoV-2 pandemic [19,20,22]. However, some variability is observed among countries. GA2.3.6b was found to be circulating in Argentina from 2019 to 2021 [21]. Our data indicate a shift in predominant circulating viruses from RSV-A to RSV-B in the 2023/2024 season, although the circulation of genotypes within RSV-A and RSV-B are similar to what was detected in previous seasons. RSV-A and RSV-B have been shown to co-circulate during epidemic seasons, with predominance altering over time [23]. The co-circulation of RSV subtypes is associated with co-detections of both RSV-A and RSV-B, comprising around 2% of cohorts in studies from the United States and Senegal [23,24]. The percentage of co-detections reported in our cohort was slightly higher; however, the clinical significance and outcomes associated with co-detections of RSV subtypes A and B are not well defined. Each genotype tends to dominate for a few consecutive seasons before being displaced by the other genotype, and the predominating genotype and length of circulation changes geographically [25,26]. Therefore, this shift to RSV-B predominance is not unexpected.

### 4.2. Analysis of the G-Gene Identifies Several Amino Acid Substitutions within RSV-A and RSV-B Samples Circulating in the JHHS

Analysis of the G-gene for RSV-A revealed 12 prominent AASs that were found across all or most of the six clades identified within the GA2.3.5 samples. All 12 AASs were found in the extracellular domain of the G-protein, within two highly glycosylated mucin-like regions (aa66-160 and aa ≈ 192–319) that are known to be highly variable [27,28]. Three mutations detected in our cohort, I134K (98.1%), S243I (100%), and K262E (98.1%), are most likely reversion mutations, often described to occur in RSV to change antigenicity, as older ON1 strains contain K134I, I243S, and E262K substitutions [1,26,29,30]. The substitutions I243S, E262K, and K134I were observed in the 2014–2015 season in China and the 2016–2017 season in Taiwan and subsequently detected in multiple countries such as Lebanon, Iran, and Italy, indicating prolonged global circulation [1,26,29,30,31,32]. An E224G was detected in an Iranian cohort during the 2018–2019 season in an epitope in escape mutants [31]. An E224V substitution was observed to have emerged in an Italian cohort, further supporting that this site is prone to substitutions resulting from immune pressure [1]. A study showed that glutamic acid (E) at position 262 was under positive selective pressure [30,33]. In our cohort, a G224E (69.2%) substitution was observed, which might also lead to antibody escape as described above. A T319I substitution was described to be circulating in Iran during the 2016–2017 season and subsequently found in 100% of samples in Lebanon in 2019, indicating global circulation of this substitution [31,34]. The Y304H and T320A substitutions, found in 82.7% and 73.1% of our samples, have been observed to be common among ON1 lineages and were the most common AASs detected in Taiwan, Lebanon, Germany, Senegal, China, Iran, and Italy [1,24,26,29,31]. Taiwanese, Lebanese, and Iranian studies reported substitutions, with differing amino acid identities, at position 320, which resulted in the loss of an N-glycosylation site [29,32,34]. In one study, the loss of this glycosylation site was identified as one of several changes thought to be responsible for a change in disease severity in Rome, Italy, during the 2016–2018 seasons [32].

Within RSV-B, seven AASs were detected across the three clades within the GB5.0.5a G-clade. All seven AASs were found in the extracellular domain of the G-protein, within two highly glycosylated mucin-like regions (aa66-160 and aa ≈ 192–319) that are known to be highly variable [27,28]. One AAS, A74V (100%), detected in our cohort was previously identified in genomes from pediatric patients in Senegal [35]. Multiple studies detected an A131T amino acid substitution within the 2016–2022 seasons across Asia and Europe, with different countries reporting its presence across various seasons [1,26]. A T137I substitution was described to often be found with the A131T substitution as well as two others, T288I and T310I, in a Chinese study during the 2016–2019 seasons [26]. In our cohort, there was a T131A (96.1%) substitution and an I137T substitution (97%), indicating a reversion back to an older strain, which has been documented within RSV to change antigenic properties to evade host antibodies [36]. A 15-year study out of Kilifi, Kenya, observed R137K/T during the 2007–2008 season where RSV-B predominated, indicating that this site has undergone past changes. In a proposal for RSV nomenclature, 137I was determined to be a defining mutation for RSV-B genotype GB1 [37]. I288T and I310T were found in only one sample, which also contained T131A and I137T. This illustrates that RSV substitutions are spatially and temporally dynamic. An S100G emerged within the 2022–2023 surveillance season in Italy, northwest Spain, and Bulgaria [1,22]. Our samples also displayed the S100G (98.5%) substitution, indicating a potential fitness advantage and global spread. This substitution has become prominent within RSV-B and is a clade-defining mutation for B5.0.5a in a novel RSV nomenclature proposal by Goya et al. [37].

### 4.3. Analysis of the F-Gene Identifies Several Amino Acid Substitutions within RSV-A and RSV-B Samples Circulating in the JHHS

The RSV F-protein is essential in the RSV viral life cycle and, along with the G-protein, promotes membrane fusion and viral entry [2,38]. It is synthesized as an inactive precursor protein, which is subsequently cleaved at two sites, aa109 and aa136, into two subunits, F1 (137-57) and F2 (aa26-109) [38]. There are six main antigenic sites within the F-protein: Ø (aa92-96, aa195-227), I (aa27-45, aa312-318, aa379-389), II (aa254-277), III (aa46-54, aa301-311, aa345-353, aa367-378), IV (aa422-471), and V (aa55-61, aa146-194, aa287-300) [23]. Similarly, there are some important cytotoxic T-lymphocyte epitopes within the fusion protein [39]. The pre- and post-cleavage forms of the F-protein have distinct as well as shared antigenic sites. Site Ø and V, which are considered the most immunogenic, are found on the pre-fusion conformation whereas sites I, II, III, and IV are shared [40]. Although the F-protein is highly conserved, the signal peptide, transmembrane domain, and antigenic site Ø have been observed to be the most variable [39].

Within RSV-A, two positions were found to have AASs across most samples: T122A (43.4%) substitution and two AASs at position 127, V127I (24.5%) and V127A (9.4%). Both positions are within the p27 portion of the F-protein. A global study from the RSV 2017–2018 seasons detected a T122A substitution within the RSV-A F-protein, indicating that within the conserved F-protein, this site has undergone changes over time [23]. Similarly, a study conducted in China and the Americas found an A122T substitution in a p27 B-cell epitope, but its impact was not fully described [26,41]. A South African study found a reversion substitution A122T paired with a known escape substitution at site 123, suggesting these amino acids may be essential for maintaining protein functionality [39]. Further studies should investigate whether the substitution at position 122 affects F-protein function. Studies from Washington State in 2022–2023 and Texas in 2017 also detected a T122A substitution, suggesting its circulation in the United States in previous seasons [13,41]. Position 122 also falls within a cytotoxic T-lymphocyte epitope [28,39].

Within RSV-B two AASs were identified: S211N, found in the antigenic site Ø at the top of the protein, was present in 96.2% of our RSV-B cohort [42]. Position 389 has substitutions S389P (93%) and S389L (4.2%), which are located in antigenic site I. The S211N substitution is within the nirsevimab binding domain [20]. This substitution has been extensively studied and has no known effect on the neutralization activity of nirsevimab. The S211N substitution has become increasingly common since 2020. Substitutions at position 389 have been detected in South Africa, Brazil, and Bulgaria, indicating that this site is prone to changes [23]. Palivizumab binds within antigenic site I, where these substitutions are located. However, it has been reported that these AASs are not predicted to impact palivizumab binding [23].

Although RSV causes a significant disease burden, there exist only a few treatment and prevention options [43]. All of these target the F-protein, which exhibits high sequence and antigenic conservation between genotypes (>90% identity), is essential in the viral life cycle, and produces high levels of cross-reactive neutralizing antibodies during natural infection, making it a valuable target [43,44]. The use of mAb therapies can increase selective pressure and facilitate antibody escape mutations [43]. To date, few AASs have been detected that impact monoclonal antibody treatments [12,43]. Only one AAS, S276N, was detected in three RSV-A samples and is known to impact palivizumab binding [12]. This substitution has been detected in South Africa, Korea, China, Iran, and Lebanon [12]. Additionally, N201S and Q209L substitutions have been observed in South Africa, the Netherlands, Korea, Brazil, and the USA, affecting nirsevimab binding [12]. Four samples contained an N201K substitution, and eight contained R209Q/K substitutions. Although these positions are known to affect antibody binding, the implications of specific amino acid identities should be further investigated. Therefore, utilizing surveillance systems to monitor and detect potential escape mutations is critical for informing public health and clinical interventions.

## 5. Conclusions

RSV is the leading cause of acute lower respiratory tract infection in children worldwide and represents a significant disease burden within the United States. This study describes the 2023/2024 RSV season in the Johns Hopkins Health System. Samples from 406 patients were collected for RSV genomic characterization, resulting in 384 sequences. The RSV-B GB5.0.5a clade was predominantly detected, indicating a shift from previous RSV-A dominated seasons within the United States. The RSV-A GA2.3.5 clade was identified to a lesser extent within our cohort. Despite RSV-A circulating to a lesser extent, patients with RSV-A detections were associated with higher admission rates. Multiple amino acid substitutions were detected within both the RSV-A and RSV-B G-protein that have been associated with changes in antigenicity. Further in vitro studies are necessary to describe the impact of each AAS on antigenicity and their potential impact on monoclonal antibody function. Similarly, when the F-gene was examined, amino acid substitutions were detected within both RSV-A and RSV-B. Some of these substitutions or their locations have previously been described to impact the binding of monoclonal antibodies. Following the approval of AREXVY, a prefusion F-protein subunit vaccine against RSV, genomic surveillance will be critical for monitoring escape mutants. Given the diversity of RSV populations within a country, local surveillance efforts are crucial for monitoring RSV activity and genomic evolution.

## Figures and Tables

**Table 1:** Description of 2023/2024 study cohort. Breathing problems include wheezing and shortness of breath. Seizures include febrile seizures. Percents for symptoms were calculated out of the total number of patients with symptoms reported in their chart. All others, unless indicated, were calculated out of the total cohort or samples with characterized genotypes.

Characteristic	Number of Patients (% Cohort)			
	Cohort	RSV B	RSV A	RSV A/B
**Cohort Size**	406	282 (69.5)	78 (19.2)	14 (3.4)
**Sex, N (%)**				
**Female**	207 (51.0)	136 (48.2)	51 (65.4)	5 (35.7)
**Male**	198 (48.8)	146 (51.8)	26 (33.3)	9 (64.3)
**Nonbinary**	1 (0.2)	0	1 (1.3)	0
**Age Group, N (%)**				
**Infants (0–11 months)**	120 (29.6)	82 (29.1)	27 (34.6)	6 (42.9)
**1–5**	183 (45.1)	133 (47.2)	28 (35.9)	7 (50.0)
**6–17**	28 (6.9)	19 (6.7)	4 (5.1)	0
**18–59**	38 (9.4)	21 (7.4)	12 (15.4)	1 (7.1)
**≥60**	37 (9.1)	27 (9.6)	7 (9.0)	0
**Clinical presentation, N (%)**				
**Symptomatic**	363 (89.4)	255 (90.4)	65 (83.3)	12 (85.7)
**Not Symptomatic**	43 (10.6)	27 (9.6)	13 (16.7)	2 (14.3)
**Fever**	105 (28.9)	71 (27.8)	19 (29.2)	2 (16.7)
**Cough**	119 (32.8)	76 (29.8)	25 (38.5)	4 (33.3)
**Breathing Problems**	98 (27.0)	74 (29)	15 (23.1)	4 (33.3)
**Congestion**	21 (5.8)	12 (4.7)	6 (9.2)	1 (8.3)
**Emesis**	20 (5.5)	16 (6.3)	3 (4.6)	0
**URI**	20 (5.5)	15 (5.9)	4 (6.2)	1 (8.3)
**Flu-like Symptoms**	11 (3.0)	8 (3.1)	1 (1.5)	0
**Chest Pain**	7 (1.9)	5 (2.0)	2 (3.1)	0
**Seizures**	1 (0.3)	0	0	0
**Comorbidity, N (%)**				
**≥1 Underlying Medical Condition**	163 (40.1)	108 (38.3)	32 (41.0)	9 (64.3)
**Hypertension**	69 (17.0)	42 (14.9)	15 (19.2)	3 (21.4)
**Lung Disease**	37 (9.1)	27 (9.6)	6 (7.7)	2 (14.3)
**Kidney Disease**	47 (11.6)	29 (10.3)	12 (15.4)	3 (21.4)
**Immunosuppression**	84 (20.7)	56 (19.9)	15 (19.2)	4 (28.6)
**Diabetes**	26 (6.4)	14 (5.0)	10 (12.8)	0
**Heart Failure**	26 (6.4)	16 (5.7)	8 (10.3)	0
**Atrial Fibrillation**	19 (4.7)	14 (5.0)	3 (3.8)	1 (7.1)
**Smoker**	16 (3.9)	7 (2.5)	6 (7.7)	0
**Cerebrovascular Disease**	22 (5.4)	16 (5.7)	6 (7.7)	0
**Cancer**	97 (23.9)	61 (21.6)	25 (32.1)	4 (28.6)
**Coronary Artery Disease**	46 (11.3)	31 (11.0)	11 (14.1)	0
**Pregnancy**	5 (1.2)	1 (0.4)	2 (2.6)	0
**Outcome, N (%)**				
**Admitted**	127 (31.3)	77 (27.3)	33 (42.3)	8 (57.1)
**ICU**	30 (7.4)	21 (7.4)	7 (9.0)	1 (7.1)
**Supplemental Oxygen**	98 (24.1)	63 (22.3)	23 (29.5)	6 (42.9)

**Table 2:** Distribution of G-clades and clades for RSV-A and RSV-B sequences obtained from the JHHS 2023 cohort.

RSV-A		
	Number of Samples (% Cohort)	%
**Total**	52 (20.4)	
**G-Clade**		
**GA2.3.5**	52	100
**Clades**		
**A.D.1**	20	38.5
**A.D.2.1**	1	1.9
**A.D.3**	10	19.2
**A.D.3.1**	2	3.8
**A.D.5.1**	4	7.7
**A.D.5.2**	15	28.8
**RSV-B**		
**Total**	203 (79.6)	
**G-Clade**		
**GB.5.0.5a**	203	100
**Clades**		
**B.D.4.1**	1	0.5
**B.D.4.1.1**	15	7.4
**B.D.E.1**	187	92.1

**Table 3:** Amino acid substitutions identified within the G-region of RSV-A and RSV-B viruses in the JHHS 2023 cohort.

RSV-A							
	Number of Samples with Substitution (%)						
**Substitution**	Total	A.D.1	A.D.2.1	A.D.3	A.D.3.1	A.D.5.1	A.D.5.2
**P71L**	52 (100)	20 (100)	1 (100)	10 (100)	2 (100)	4 (100)	15 (100)
**H90Y**	50 (96.2)	20 (100)	0	9 (90.0)	2 (100)	4 (100)	15 (100)
**L101F**	46 (88.5)	18 (90.0)	1 (100)	8 (80.0)	0	4 (100)	15 (100)
**I134K**	51 (98.1)	20 (100)	0	10 (100)	2 (100)	4 (100)	15 (100)
**G224E**	36 (69.2)	2 (15.0) G224V: 17 (85.0)	1 (100)	10 (100)	2 (100)	4 (100)	15 (100)
**S243I**	52 (100)	20 (100)	1 (100)	8 (80.0)	2 (100)	4 (100)	15 (100)
**K262E**	51 (98.1)	20 (100)	0	10 (100)	2 (100)	4 (100)	15 (100)
**I265L**	52 (100)	20 (100)	1 (100)	10 (100)	2 (100)	4 (100)	15 (100)
**D284G**	50 (96.2)	20 (100)	1 (100)	9 (90.0)	1 (50)	4 (100)	15 (100)
**Y304H**	43 (82.7)	20 (100)	1 (100)	2 (20.0)	1 (50)	4 (100)	15 (100)
**T319I**	36 (69.2)	17 (85.0)	T319S1 (100)	0	0	4 (100)	15 (100)
**T320A**	38 (73.1)	18 (90.0)	0	0	1 (50)	4 (100)	15 (100)
**RSV-B**							
	Number of Samples with Substitution (%)						
**Substitution**	Total	B.D.4.1	B.D.4.1.1	B.D.E.1			
**A74V**	203 (100)	1 (100)	15 (100)	187 (100)			
**T131A**	195 (96.1)	1 (100)	13 (86.7)	181 (96.8)			
**I137T**	197 (97.0)	1 (100)	13 (86.7)	183 (97.9)			
**I252T**	184 (90.6)	1 (100)	1 (6.7)	182 (97.3)			
**P289L**	5 (2.5)	1 (100)	1 (6.7)	3 (1.6)			
**S100G**	200 (98.5)	0	13 (86.7)	187 (100)			
**P221L**	196 (96.6)	0	12 (80.0)	184 (98.4)			

**Table 4:** AASs identified within the F-region of RSV-A and RSV-B viruses in the JHHS 2023/2024 cohort.

RSV-A		
**Total**	53	
**Substitution**	Number of Samples with Substitution	%
**T122A**	23	43.4
**V127I**	13	24.5
**V127A**	5	9.4
**RSV-B**		
**Total**	213	
**Substitution**	Number of Samples with Substitution	%
**S211N**	205	96.2
**S389P**	198	93.0
**S389L**	9	4.2

**Table 5:** Amino acid substitutions in RSV-A and RSV-B F-region known to or * at positions known to impact monoclonal antibody binding.

Substitution	Number of Samples	%	Monoclonal Antibody Impacted
**RSV-A**			
**S276N**	3	5.7 [3/53]	Palivizumab
**RSV-B**			
N201K *	4	1.9 [4/213]	Nirsevimab
R209Q *	5	2.3 [5/213]	Nirsevimab
R209K *	3	1.4 [3/213]	Nirsevimab

## Data Availability

G and F fasta files are attached as Supplementary datasets 1–4.

## Supplementary Materials

The following supporting information can be downloaded at https://www.mdpi.com/article/10.3390/v16071122/s1, Table S1: Reference sequences used for the phylogenetic analysis for RSV-A and RSV-B. Table S2: Patient metadata stratified by age group. Supplementary datasets 1–4: fasta files of RSV-A G, RSV-B G, RSV-A F, and RSV-B F-genes.
