# Supplementary material for: Genomic Evolution and Surveillance of Respiratory Syncytial Virus during the 2023–2024 Season
Source: Viruses. 2024 Jul 12;16(7):1122. doi: 10.3390/v16071122 (PMC11281595; doi:10.3390/v16071122)
Supplement: Supplementary file 1 [file viruses-16-01122-s001.zip › Table S1.pdf]

Supplementary Table 1: Reference sequences used for the phylogenetic analysis for RSV-A and RSV-B. Country and year indicate where and when the patient samples were collected.

RSV-A G-gene					
Sequence ID	Accession Number	Genotype	Clade	Country	Year
MN-MDH-RSVA-00028	PP135024.1	GA2.3.5	A.D.3	USA	2023
RSVA/20190508/BJ/CHN/2019.04.19	OR666534.1	GA2.3.5	A.D.3	China	2019
RSV/Human/USA/MA-Broad_MGB-13671/2022	OQ024150.1	GA2.3.5	A.D.3	USA	2022
RSV-A/human/USA/IL-NM-RSV410/2022	PP352329.1	GA2.3.5	A.D.5.1	USA	2022
hRSV/A/Germany/22-03065/2022	OR795471.1	GA2.3.5	A.D.5.2	Germany	2022
hRSV-A-HCV11932	OR915768.1	GA2.3.5	A.D.5.2	USA	2022
hRSV/A/USA/2022LTGQ4/2022	OP890316.1	GA2.3.5	A.D.2.1	USA	2022
RSV-A/human/USA/IL-NM-RSV443/2022	PP352347.1	GA2.3.5	A.D.1	USA	2022
ASU102704	OR143171.1	GA2.3.5	A.D.1	USA	2023
hRSV/A/England/397/2017	EPI_ISL_412866	GA2.3.2b	A.3	England	2017
hRSV/A/W13J93V3	OR872604.1	GA2.3.5	A.D.3.1	USA	2022
RSVA/Leipzig/2021/39-1	OP927870.1	GA2.3.5	A.D.5.2	Germany	2021
hRSV/A/England/397/2017	EPI_ISL_412866	GA2.3.2b	A.3		
RSV-B G-gene					
Sequence ID	Accession Number	Genotype	Clade		
RSV/Human/USA/MA-Broad_MGB-13787/2022	OQ171934.1	GB5.0.5a	B.D.E.1	USA	2022
hRSV-B-G43Q53R8	OR915810.1	GB5.0.5a	B.D.E.1	USA	2023

RSV-B/human/USA/WA-S25047/2024	PP495970.1	GB5.0.5a	B.D.E.1	USA	2024
RSV-B/human/USA/WA-S25082/2021	PP496021.1	GB5.0.5a	B.D.E.1	USA	2021
MN-MDH-RSVB-00045	PP237797.1	GB5.0.5a	B.D.E.1	USA	2023
RSV-B/human/USA/WA-S23481/2021	OR326833.1	GB5.0.5a	B.D.E.1	USA	2021
PEH231032	PP411987.1	GB5.0.5a	B.D.E.1	Thailand	2023
PEH230734	PP411978.1	GB5.0.5a	B.D.4.1.1	Thailand	2023
Patient_F_D0	OK500263.1	GB5.0.5a	B.D.4.1	France	2021
RSV-B/human/USA-IL-NM-RSV335/2021	PP352454.1	GB5.0.5a	B.D.E.1	USA	2021
RSV-B/human/USA/WA-S24500/2023	PP084064.1	GB5.0.5a	B.D.E.1	USA	2023
RSV-B/human/USA/WA-S24717/2023	PP342457.1	GB5.0.5a	B.D.E.1	USA	2023
MN-MDH-RSVB-00039	PP135035.1	GB5.0.5a	B.D.E.1	USA	2023
hRSV/B/Australiza/VIC-RCH056/2019	EPI_ISL_1653999	GB5.0.3	B.D.2	Australia	2019
RSV-B/human/USA/IL-NM-RSV520/2022	PP352492.1	GB5.0.5a	B.D.E.1	USA	2022
RSV-B/human/USA/WA-S24522/2023	PP084086.1	GB5.0.5a	B.D.E.1	USA	2023
RSV-B/human/USA/WA-S24746/2023	PP342486.1	GB5.0.5a	B.D.E.1	USA	2023
RSV-B/human/USA/IL-NM-RSV137/2018	PP352404.1	GB5.0.5a	B.D.4.1.1	USA	2018

hRSV/B/Germany/22-01678/2021	OR795255.1	GB5.0.5a	B.D.E.1	Germany	2021
hRSV/B/Australiza/VIC-RCH056/2019	EPI_ISL_1653999	GB5.0.3	B.D.2		
ASU102752	OR143242.1	GB5.0.5a	B.D.E.1	USA	2022
