# Supplementary material for: Genomic Evolution and Surveillance of Respiratory Syncytial Virus during the 2023–2024 Season
Source: Viruses. 2024 Jul 12;16(7):1122. doi: 10.3390/v16071122 (PMC11281595; doi:10.3390/v16071122)
Supplement: Supplementary file 1 [file viruses-16-01122-s001.zip › Table S2.pdf]

Supplementary Table 2: Patient comorbidities and severity stratified by age and genotype as indicated by patient chart review.

**Cohort Total** (% cohort)					
**Age groups**	Infants (0-11 months)	1-5	6-17	18-59	60+
**Comorbidities**, N (SD)					
≥1 underlying medical condition	19 (15.8)	58 (31.7)	16 (57.1)	33 (86.8)	37 (100)
Hypertension	2 (1.7)	14 (7.7)	7 (25.0)	12 (31.6)	34 (91.9)
Lung Disease	0	13 (7.1)	5 (17.9)	6 (15.8)	13 (35.1)
Kidney Disease	3 (2.5)	5 (2.7)	4 (14.3)	11 (28.9)	24 (64.9)
Immunosuppression	6 (5.0)	22 (12.0)	9 (32.1)	18 (47.4)	29 (78.4)
Diabetes	0	0	0	10 (26.3)	16 (43.2)
Heart Failure	1 (0.8)	0	0	7 (18.4)	18 (48.6)
Atrial Fibrillation	0	0	0	3 (7.9)	16 (43.2)
Smoker	0	0	0	7 (18.4)	9 (24.3)
Cerebrovascular Disease	2 (1.7)	5 (2.7)	0	4 (10.5)	11 (29.7)
Cancer	8 (6.7)	25 (13.7)	7 (25.0)	22 (57.9)	35 (94.6)
Coronary Artery Disease	0	1 (0.5)	2 (7.1)	10 (26.3)	33 (89.2)
Pregnancy	0	0	0	5 (13.2)	0
**Outcome,** N (SD)					
Admitted	32 (26.7)	38 (20.8)	12 (42.9)	17 (44.7)	28 (75.7)
ICU	9 (7.5)	7 (3.8)	4 (14.3)	5 (13.2)	5 (13.5)
Supplemental Oxygen	33 (27.5)	29 (15.8)	9 (32.1)	9 (23.7)	18 (48.6)
**RSV-B** (% cohort)					
**Age groups**	Infants (0-11 months)	1-5	6-17	18-59	60+
**Comorbidities**, N (SD)					
≥1 underlying medical condition	12 (14.6)	43 (32.3)	9 (47.4)	17 (81.0)	27 (100)
Hypertension	1 (1.2)	7 (5.3)	4 (21.1)	4 (19.0)	26 (96.3)
Lung Disease	0	11 (8.3)	3 (15.8)	4 (19.0)	9 (33.3)
Kidney Disease	3 (3.7)	1 (0.8)	3 (15.8)	4 (19.0)	18 (66.7)
Immunosuppression	3 (3.7)	16 (12.0)	6 (31.6)	8 (38.1)	23 (85.2)
Diabetes	0	0	0	3 (14.3)	11 (40.7)
Heart Failure	0	0	0	2 (9.5)	14 (51.9)
Atrial Fibrillation	0	0	0	1 (4.8)	13 (48.1)
Smoker	0	0	0	1 (4.8)	6 (22.2)
Cerebrovascular Disease	2 (2.4)	3 (2.3)	0	3 (14.3)	8 (29.6)
Cancer	5 (6.1)	17 (12.8)	3 (15.8)	11 (52.4)	25 (92.6)
Coronary Artery Disease	0	1 (0.8)	2 (10.5)	3 (14.3)	25 (92.6)
Pregnancy	0	0	0	3 (14.3)	0
**Outcome,** N (SD)					
Admitted	18 (22.0)	21 (15.8)	9 (47.4)	8 (38.1)	21 (77.8)
ICU	6 (7.3)	5 (3.8)	4 (21.1)	1 (4.8)	5 (18.5)
Supplemental Oxygen	20 (24.4)	17 (12.8)	8 (42.1)	4 (19.0)	14 (51.9)
**RSV-A** (% cohort)					

**Age groups**	Infants (0-11 months)	1-5	6-17	18-59	60+
**Comorbidities**, N (SD)					
≥1 underlying medical condition	2 (7.4)	8 (28.6)	3 (75.0)	12 (100)	7 (100)
Hypertension	0	3 (10.7)	1 (25.0)	6 (50.0)	5 (71.4)
Lung Disease	0	0	1 (25.0)	2 (16.7)	3 (42.9)
Kidney Disease	0	1 (3.6)	1 (25.0)	6 (50.0)	4 (57.1)
Immunosuppression	1 (3.7)	2 (7.1)	1 (25.0)	8 (66.7)	3 (42.9)
Diabetes	0	0	0	7 (58.3)	3 (42.9)
Heart Failure	1 (3.7)	0	0	5 (41.7)	2 (28.6)
Atrial Fibrillation	0	0	0	1 (8.3)	2 (28.6)
Smoker	0	0	0	4 (33.3)	2 (28.6)
Cerebrovascular Disease	0	2 (7.1)	0	1 (8.3)	3 (42.9)
Cancer	1 (3.7)	4 (14.3)	3 (75.0)	10 (83.3)	7 (100)
Coronary Artery Disease	0	0	0	6 (50.0)	5 (71.4)
Pregnancy	0	0	0	2 (16.7)	0
**Outcome,** N (SD)					
Admitted	9 (33.3)	11 (39.3)	1 (25.0)	8 (66.7)	4 (57.1)
ICU	3 (11.1)	0	0	4 (33.3)	0
Supplemental Oxygen	9 (33.3)	7 (25.0)	1 (25.0)	4 (33.3)	2 (28.6)
**RSV-A/B** (% cohort)					
**Age groups**	Infants (0-11 months)	1-5	6-17	18-59	60+
**Comorbidities**, N (SD)					
≥1 underlying medical condition					
Hypertension	4 (66.7)	4 (57.1)	0	1 (100)	0
Lung Disease	0	2 (28.6)	0	1 (100)	0
Kidney Disease	0	2 (28.6)	0	0	0
Immunosuppression	0	2 (28.6)	0	1 (100)	0
Diabetes	1 (16.7)	3 (42.9)	0	0	0
Heart Failure	0	0	0	0	0
Atrial Fibrillation	0	0	0	0	0
Smoker	0	0	0	1 (100)	0
Cerebrovascular Disease	0	0	0	0	0
Cancer	2 (33.3)	2 (28.6)	0	0	0
Coronary Artery Disease	0	0	0	0	0
Pregnancy	0	0	0	0	0
**Outcome,** N (SD)					
Admitted	4 (66.7)	3 (42.9)	0	1 (100)	0
ICU	0	1 (14.3)	0	0	0
Supplemental Oxygen	3 (50.0)	2 (28.6)	0	1 (100)	0
